# Interleukin-1*β* Enhances Umbilical Cord Mesenchymal Stem Cell Adhesion Ability on Human Umbilical Vein Endothelial Cells via LFA-1/ICAM-1 Interaction

**DOI:** 10.1155/2019/7267142

**Published:** 2019-12-27

**Authors:** Tsai-Yi Wu, Ya-Han Liang, Jiahn-Chun Wu, Hwai-Shi Wang

**Affiliations:** Institute of Anatomy and Cell Biology, School of Medicine, National Yang Ming University, Taipei, Taiwan

## Abstract

The migration of administered mesenchymal stem cells (MSCs) to sites of injury via the bloodstream has been demonstrated. However, the underlying mechanisms of umbilical cord MSC adhesion to endothelial cells during transendothelial migration are still unclear. In this study, our data showed that IL-1*β* induced LFA-1 expression on MSCs and ICAM-1 expression on HUVECs. We then pretreated MSCs with protein synthesis inhibitor cycloheximide. The results showed that IL-1*β* induced LFA-1 expression on the surface of MSCs via the protein synthesis pathway. Through the p38 MAPK signaling pathway inhibitor SB 203580, we found that IL-1*β* induces the expression of LFA-1 through p38 MAPK signaling and enhances ICAM-1 expression in HUVECs. In addition, IL-1*β*-induced MSC adhesion to HUVECs was found to be inhibited by IL-1RA and the LFA-1 inhibitor lovastatin. These results indicate that IL-1*β* promotes the cell adhesion of MSCs to HUVECs through LFA-1/ICAM-1 interaction. We address the evidence that the cell adhesion mechanism of IL-1*β* promotes MSC adhesion to HUVECs. The implications of these findings could enhance the therapeutic potential of MSCs.

## 1. Introduction

Umbilical cord mesenchymal stem cells (UC-MSCs) are multipotent cells with the capacity for self-renewal and differentiation into cells of the cardiomyogenic, adipogenic, and osteogenic lineages [1]. MSCs also have the ability to secrete paracrine factors and to home in on sites of inflammation following tissue injury in a mouse model [2–4]. Previous research has shown that treatment strategies such as pretreatment with cytokines or growth factors may improve MSC migration and adhesion [5, 6]. Although preclinical and clinical evidence of therapeutic benefit of MSCs in various medical conditions has been substantiated, one major obstacle in MSC therapeutic is harsh microenvironments that interfere with the MSC homing ability and obscures our knowledge of the initial step of cell adhesion mechanism during transendothelial migration.

IL-1*β* is a highly inflammatory cytokine produced when tissue is inflamed due to the presence of monocytes and macrophages [7]. They are secreted and circulated systemically [8]. In our previous study, we found that interleukin-1*β* (IL-1*β*) induced mesenchymal stem cell migration in vitro [9, 10]. IL-1𝛽 pretreatment enhanced the efficacy of MSC transplantation in dextran sulfate sodium- (DSS-) induced colitis [11]. It has been shown that IL-1*β* upregulates the expression of many genes including cytokines and adhesion molecules [12]. IL-1*β* induced ICAM-1 expression in human umbilical vein endothelial cell (HUVEC) [13], ICAM-1, and VCAM-1 expression in human vascular smooth muscle cells [14].

Cell-to-cell and cell-to-matrix interactions that are critical to cell migration, growth, and survival are largely mediated by integrins [15]. The integrin LFA-1/ICAM-1interaction has been considered one of the major pairs of adhesion molecules contributing to the different steps of leukocyte migration across the endothelium [16]. Research has shown that leukocyte adhesion during inflammation proceeds in a cascade-like fashion, in which integrins are responsible for leukocyte firm adhesion and transmigration [17]. There is evidence that MSCs pass through capillaries to postcapillary venules in a manner similar to leukocyte homing [18]. Although ICAM-1 expression on endothelial cells has been implicated in active MSCs, it is still not known which ligands are present in MSC interaction with ICAM-1.

Lymphocyte function-associated antigen 1 (LFA-1) is an *α*L*β*2 heterodimeric integrin composed of two chains, CD11a and CD18. It plays an important role in immune cell adhesion and migration [19]. The main ligand of LFA-1 is intercellular adhesion molecule-1 (ICAM-1) [20, 21]. LFA-1 and ICAM-1 interaction plays a role in many immunological response processes including adhesion and transmigration of leukocytes through the endothelium [17]. Previous studies have shown that coculture of human smooth muscle cell with MSCs increases VCAM-1-dependent migration and that, during this process, LFA-1 plays an important role in MSC migration [22]. Many studies have demonstrated that LFA-1 expression can be enhanced by cytokines such as IL-1, TNF-*α*, and TGF-*β* [23, 24]. It has been found that both IL-1*β* and LFA-1 are highly expressed in rat chronic esophagitis [25]. Recent studies found that MSCs pretreated with kinase inhibitor Ro-31-8425 enhance CD11a expression and induce firm adhesion of MSCs to ICAM-1 [26].

The IL-1 signaling pathway has already shown that IL-1*β*-induced IL-1R will activate mitogen-activated protein kinase (MAPK) cascades [27]. MAPK have three members including extracellular signal-regulated kinase1/2 (ERK1/2), c-JUN N-terminal kinases (JNK), and p38 [28]. It has been shown that IL-1*β* alone can activate p38, ERK1/2, and JNK1/2 in osteoblastic cells [29]. Furthermore, IL-1*β* regulation of cell-base adhesion between astrocytes and the extracellular matrix has been proven through cross-talk mechanisms between ERK1/2 and inhibition of RhoA and Rho kinase [30]. Another study showed that IL-1*β* activates the p38 MAPK signaling pathway and enhances cell migration ability in renal proximal tubular cells [31].

In this study, we show that MSC adhesion to HUVECs is induced by IL-1*β*. Following results of cited references, we predict that LFA-1/ICAM-1 conduct MSC-HUVEC-adhesive interactions. We investigated the IL-1 pathway and examined in particular the role of p38 MAPK induced by IL-1*β* involved in IL-1*β*-induced LFA-1 expression. Our investigation found that IL-1*β*-mediated MSC adhesion to HUVECs depends on LFA-1/ICAM-1 expression, which involves p38 MAPK signaling transduction pathway in LFA-1 expression.

## 2. Materials and Methods

### 2.1. Cell Culture

Umbilical cord mesenchymal stem cells (UC-MSCs) were purchased from the Bioresource Collection and Research Center, Hsinchu, Taiwan. The culturing condition was prepared using previously described methods [1]. Mesenchymal stem cells were cultured in a low glucose-defined medium consisting of 56% low-glucose Dulbecco's modified Eagle medium (DMEM; Invitrogen, CA, USA), 37% MCBD 201 (Sigma, MO, USA), 2% fetal bovine serum (Thermo, Logan, UT), 0.5 mg/ml of AlbuMAX® I (Invitrogen, CA, USA), 50 nM L-ascorbic acid 2-phosphate (Sigma, MO, USA), 10 nM dexamethasone (Sigma, MO, USA), 1x antibiotic antimycotic solution (Thermo, Logan, UT), 1x insulin-transferrin-selenium-A (Invitrogen, CA, USA), 10 ng/ml of epidermal growth factor (PeproTech, NJ, USA), and 1 ng/ml of platelet-derived growth factor-BB (PeproTech, NJ, USA) at 37°C and 5% CO_2_. When cells reached 70-80% confluence, they were detached using HyQTase (Thermo, Logan, UT) and reseeded at a ratio of 1 : 4.

Human umbilical vein endothelial cells (HUVECs) from human umbilical cords were obtained from full-term births with mother's consent. The methods to isolate HUVECs follow the Chen et al. method [32]. HUVECs were cultured on 1% gelatin (Sigma, MO, USA) and maintained in homemade medium consisting of 98% DMEM/F12, 2% fetal bovine serum (Thermo, Logan, UT), 1 *μ*g/ml hydrocortisone (Sigma, MO, USA), 20 *μ*g/ml heparin sulfate (Sigma, MO, USA), 250 ng/ml insulin (Sigma, MO, USA), 1x penicillin-streptomycin solution (Thermo, Logan, UT), 5 ng/ml of epidermal growth factor (PeproTech, NJ, USA), and 10 ng/ml fibroblast growth factor-basic (PeproTech, NJ, USA), at 37°C and 5% CO_2_. When cells reached 70-80% confluence, they were detached using HyQTase (Thermo, Logan, UT) and reseeded at a ratio of 1 : 3. Passages 3 to 4 were used in all the experiments.

### 2.2. Cytokines and Inhibitors

MSCs were starved for 15-18 hours in serum-free DMEM/LG containing 0.5% fetal bovine serum (Thermo, Logan, UT), then treated with 2 *μ*g/ml IL-1*β* inhibitor IL-1RA (PeproTech, NJ, USA) for 2 hours prior to cytokine stimulation. The LFA-1/ICAM-1 inhibitor lovastatin (Cayman Chemical, USA) was added to the cell coculture at a concentration of 50 *μ*M. MAPK p38 inhibitor SB 203580 (5 *μ*M) (Tocris, UK) was added to the cell culture after IL-1*β* stimulation. According to our previous study, MSCs treated with 100 ng/ml human recombinant IL-1*β* for 18 hours significantly enhanced migration without affecting cell viability and cell proliferation [10, 32]. At the indicated time, cells were incubated for 30 minutes with 100 ng/ml human recombinant IL-1*β* (PeproTech, NJ, USA) in the continued presence of these inhibitors.

HUVECs were starved for 3 hours in serum-free DMEM/F12 containing 1% bovine serum albumin (Sigma, MO, USA), then treated with 100 ng/ml IL-1*β* for 6 hours.

### 2.3. Cell Viability Assay

Cells were plated in 96-well plates in serum-free DMEM containing 0.5% FBS for 15-18 hours and stimulated with 100 ng/ml human recombinant interleukin-1*β* for 30 minutes. MTT assay reagent (3-(4,5-dimethyl-2-thiazolyl)-2,5-diphenyl-2H-tetrazolium bromide) (SERVA, Heidelberg, German) was added directly to the culture medium, and the cells were incubated for 4 hours at 37°C. DMSO was then added to the cells (Sigma, MO, USA) for 2 hours. The results were detected using multimode microplate readers (Infinite 200, TECAN) under absorbance of 545 nm.

### 2.4. Western Blotting

To separate cytosolic and membrane-associated protein fractions, cells were treated using a Mem-PER™ Plus Membrane Protein Extraction Kit (Thermo, IL, USA) with a Halt Protease Inhibitor Cocktail (Thermo, IL, USA). First, cells were washed and scraped with PBS. The cell suspension was then centrifuged at 300 g for 5 minutes. Cell pellets were washed with a cell wash solution and centrifuged at 300 g for 5 minutes. Second, permeabilization buffer with 1% protease inhibitor was added to the cell pellets and incubated for 30 minutes at 4°C followed by centrifugation at 16,000 g for 15 minutes at 4°C to collect the supernatant containing the cytosolic protein. To harvest the membrane protein, we added a solubilization buffer with 1% protease inhibitor to the pellets and incubated for 60 minutes at 4°C, then centrifugation at 16,000 g for 15 min at 4°C to collect the supernatant containing solubilized membrane and membrane-associated proteins. To prepare the whole cell lysate, cells were washed with PBS and lysed using an M-PER mammalian protein extraction reagent (Thermo, IL, USA) with a Halt Protease Inhibitor Cocktail (Thermo, IL, USA) followed by centrifugation at 14,000 g for 10 minutes at 4°C to collect the precleared cell extracts. Protein concentration was determined with the Coomassie Plus (Bradford) protein assay reagent (Thermo, IL, USA) using multimode microplate readers (Infinite 200, TECAN). Protein samples were resolved by 8% sodium dodecyl sulfate-polyacrylamide gel electrophoresis (SDS-PAGE) and transferred to polyvinylidene fluoride membranes (Merck, Darmstadt, Germany). The membrane was blocked in 5% fish gelatin blocking buffer (Amresco, OH, USA) for 1 hour and then incubated with the anti-human CD11a antibody (GeneTex, USA) at 1 : 4000 dilution, anti-p38 MAPK antibody (Santa Cruz, TX, USA) at 1 : 200 dilution, and phospho-p38 MAPK primary antibodies (Cell Signaling, MA, USA) at 4°C overnight. The blots were washed with Tris-buffered saline with Tween 20 (TBST) and incubated with goat anti-rabbit secondary antibody for 1 hour at room temperature. Membranes were washed and then detected by an enhanced chemiluminescence substrate using the Luminescence Imaging System (LAS-4000, GE, USA).

### 2.5. Cell Immunofluorescence and Image

Cells were plated in microscope cover glasses (12 mm) for 2 days, incubated in starvation medium for 16 hours, and then stimulated with interleukin-1*β* at different times. Cells were fixed in 4% paraformaldehyde (Ferak Berlin GmbH, German) for 15 min. Cells were then blocked with 2% bovine serum albumin (BSA, Sigma, MO, USA) and then incubated with the anti-human CD11a antibody (GeneTex, USA) at 1 : 200 dilution or ICAM-1 antibody (R&D system, USA) at 1 : 100 dilution at 4°C overnight. The blots were washed with PBS and incubated with rabbit anti-mouse secondary antibody or mouse anti-rabbit secondary antibody (1 : 200) for 1 hour at room temperature. After washing three times with PBS, cells were stained using Hoechst 33258 (Sigma, MO, USA) at 1 : 5000 dilution to identify cell nucleus and mounted with a Fluorescence Mounting Medium (Dako, CA, USA). Images of cells were acquired using a laser confocal microscope (FV1000, Olympus).

### 2.6. Immunofluorescence Study Adhesion Assay

HUVECs at passage 3-4 were seeded on a 24-well plate. After 3-4 days, a confluent monolayer was formed, then starved 3 hours and treated with IL-1*β* for 6 hours. MSCs were labeled with calcein AM (Tocris, UK) 6 *μ*M. Then, MSCs (4 × 10^4^/900 *μ*l) were cocultured with HUVECs for 30 minutes in DMEM/F12 medium with 1% BSA at 37°C. After adhesion, PBS with Ca^2+^/Mg^2+^ was used to wash cells 3 times in order to wash out nonadhesion cells. Cells were stained using Hoechst 33258 at 1 : 3000 dilutions to identify the cell nucleus, fixed in 4% paraformaldehyde for 15 minutes, and then mounted with a Fluorescence Mounting Medium. Images of cells were acquired by using a fluorescence microscopy (DM6000B, Leica). The cell number was counted at 10x magnification (five random fields of view).

### 2.7. Statistical Analysis

Statistical analyses were performed using Prism 5 software. Quantitation data were analyzed by Student's *t*-test and one-way ANOVA. *P* values < 0.05 were considered statistically significant.

## 3. Results

### 3.1. IL-1*β* Stimulates LFA-1 Expression in MSCs

**Figure 1:**
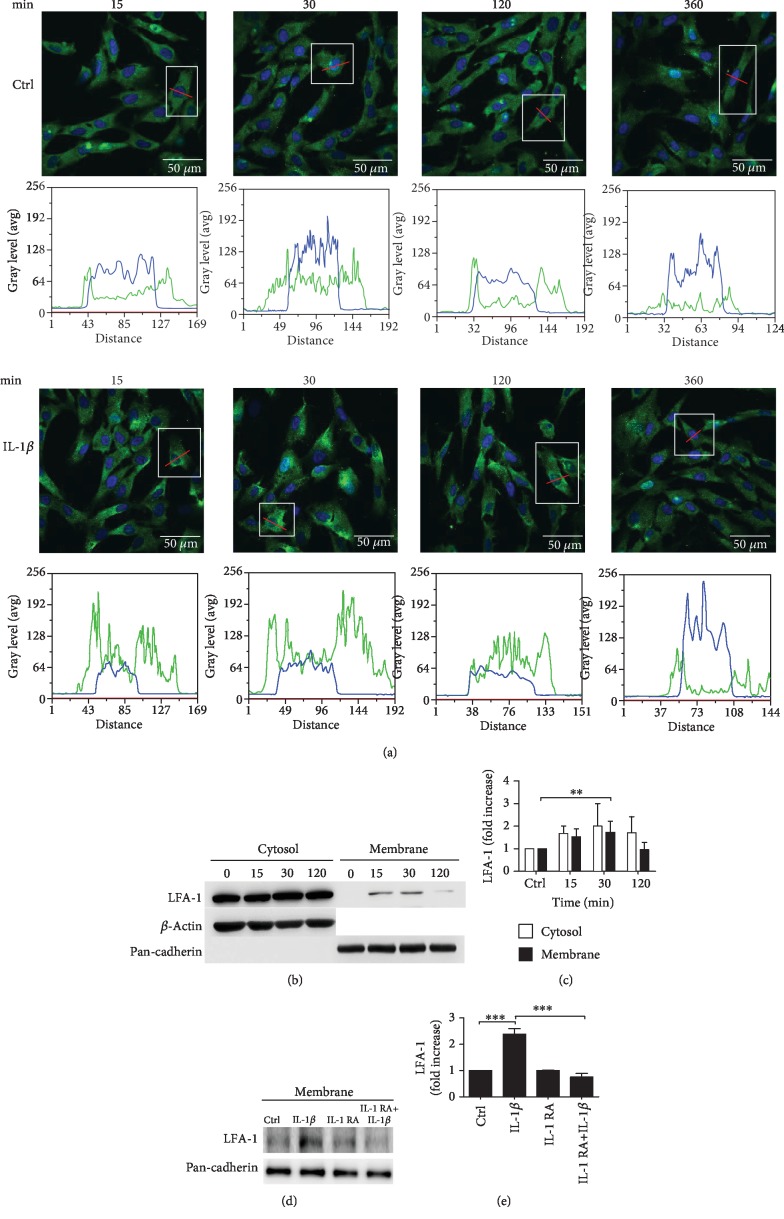
IL-1*β* induces increased expression of LFA-1 in MSCs. (a) MSCs without treated IL-1*β* as a control group and treated with IL-1*β* for 15, 30, 120, and 360 minutes (scale bar: 50 *μ*m). Histograms analyze LFA-1 (green) fluorescence intensity and location of one single MSC. White blocks show a single MSC. Red lines show the histogram detection sites. Results of measurement of LFA-1 (green) and cell nucleus (blue) fluorescence intensity are quantified by MetaMorph. (b) Example of Western blot results of the LFA-1 (129 kDa) expression in cytosolic and membrane fractions. Cells were treated with IL-1*β* for 15, 30, and 120 minutes. (c) Quantitative graphs of the Western blot results of LFA-1 protein expression of (b) (*n* = 3, ^∗∗^*P* < 0.01). (d) Example of Western blot results of the LFA-1 (129 kDa) from cell membrane protein. Cells treated with IL-1*β* and IL-1RA or cotreated with IL-1*β* and IL-1RA. (e) Quantitative graphs of the Western blot results of LFA-1 protein expression of (d) (*n* = 3, ^∗∗∗^*P* < 0.005, and ^∗∗^*P* < 0.01).

To determine whether IL-1*β* stimulates LFA-1 expression level on MSC cell membrane, immunocytochemistry staining was used to analyze fluorescence intensity. MSCs were treated with 100 ng/ml IL-1*β* for 15, 30, 120, and 360 minutes to induce LFA-1 expression. Results showed that IL-1*β* induced LFA-1 expression to the highest level at 30 minutes. To further confirm LFA-1 expression, we used histograms to analyze the data. As shown in Figure 1(a), the fluorescence intensity of LFA-1 greatly increased when MSCs were treated with IL-1*β* for 30 minutes in comparison to those of other groups. The cell viability assay indicated no significant change after IL-1*β* treatment for 30 minutes in comparison to the control group (Suppl. Fig. [Supplementary-material supplementary-material-1]).

To investigate whether IL-1*β* induces LFA-1 protein expression in MSCs, we treated MSCs with IL-1*β* (100 ng/ml) for 15, 30, and 120 minutes. Total proteins were extracted and membrane proteins were then separated. We used Western blotting to analyze LFA-1 protein expression (Figures 1(b) and 1(c)). Results demonstrated that LFA-1 protein expression was significantly upregulated after IL-1*β* treatment for 30 minutes on cell membranes.

To further confirm whether IL-1*β* could induce LFA-1 protein expression in MSC cell membranes, Western blotting analysis was performed. MSCs were pretreated with IL-1*β* inhibitor IL-1RA (2 *μ*g/ml) for 2 hours, then cotreated with IL-1*β* and IL-1RA for 30 minutes. Results demonstrated the inhibitor significantly suppressed IL-1*β*-induced LFA-1 protein expression (Figures 1(d) and 1(e)). To clarify whether treatment with IL-1RA may impact cell viability, a cell viability assay was performed and the results showed no significant change after IL-1RA treatment for 150 minutes when compared to the control group (Suppl. Fig. [Supplementary-material supplementary-material-1]).

### 3.2. Effects of IL-1*β* Induce MSC Adhesion Ability to HUVECs by LFA-1/ICAM-1 Interaction

**Figure 2:**
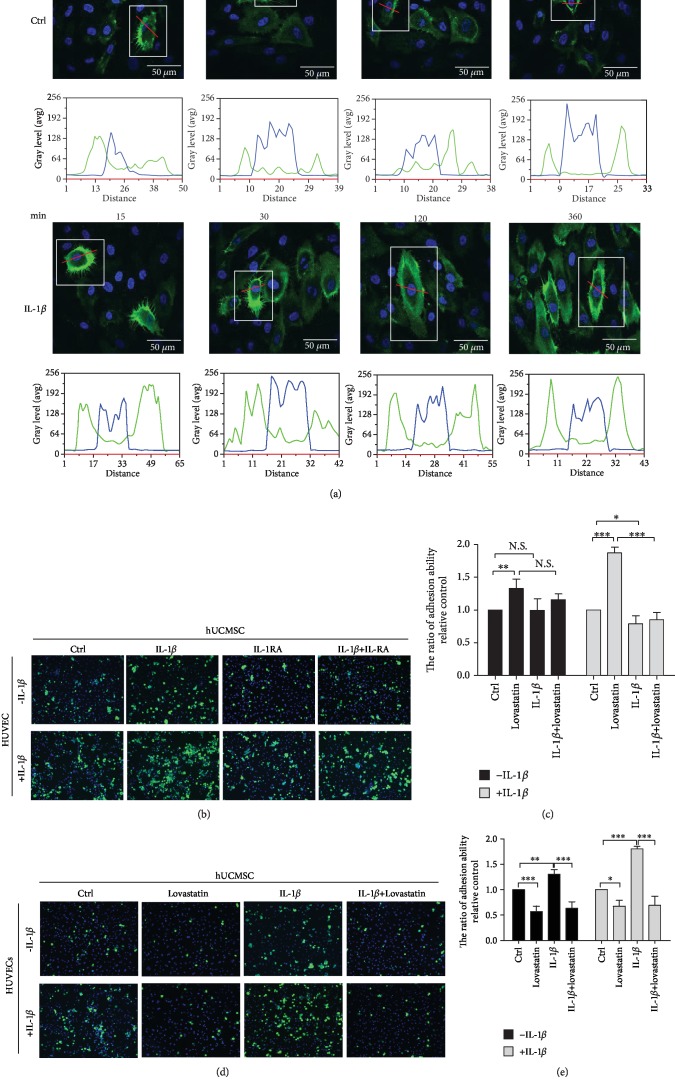
IL-1*β*-induced ICAM-1 expression in HUVECs and enhanced MSC adhesion to HUVECs via LFA-1/ICAM-1. (a) Immunocytochemistry staining of ICAM-1 (green) and cell nucleus (blue) in HUVECs treated with or without IL-1*β* for 15, 30, 120, and 360 minutes (scale bar: 50 *μ*m). Histograms analyze ICAM-1 (green) fluorescence intensity and location of one single MSC. White blocks show a single MSC. Red lines show histogram detection sites. Results of measured ICAM-1 (green) and cell nucleus (blue) fluorescence intensity are quantified by MetaMorph. (b) Representative image of the adhesion of MSCs to HUVECs. MSCs were treated with IL-1*β*, IL-1RA, and adhesion to IL-1*β*-activated HUVECs or nonactivated HUVECs. MSCs labelled with calcein AM (5 *μ*M) (green), HUVECs, and MSC cell nuclei were stained with Hoechst 33258 (blue) (scale bar: 50 *μ*m). (c) Quantitative graphs of the cell adhesion assay results of MSC adhesion to nonactivated HUVECs (black bars) or IL-1*β*-activated HUVECs (gray bars). Values were the cell number fold change relative to the control group. Data represent mean ± SD (^∗∗∗^*P* < 0.005, ^∗∗^*P* < 0.01, and ^∗^*P* < 0.05) (N.S.: nonsignificance). (d) Representative image of MSC adhesion on HUVECs. Cell adhesion assay to determine the percentage of MSC adhesion on the HUVEC monolayer. MSC adhesion to IL-1*β*-activated HUVECs or nonactivated HUVECs after treatment with IL-1*β* and the inhibitor lovastatin. (e) Quantitative graphs of the cell adhesion assay results of MSC adhesion to nonactivated HUVECs (black bars) or IL-1*β*-activated HUVECs (gray bars). MSCs treated with IL-1*β* and the inhibitor lovastatin. Data represent mean ± SD (*n* = 3,^∗∗∗^*P* < 0.005, ^∗∗^*P* < 0.01, and ^∗^*P* < 0.05).

To identify IL-1*β*-stimulated ICAM-1 expression level on cell membranes of HUVECs, immunocytochemistry staining was used to analyze cell protein location and fluorescence intensity. HUVECs were treated with 100 ng/ml IL-1*β* for 15, 30, 120, and 360 minutes to induce ICAM-1 expression (Figure 2(a)). Results showed that IL-1*β* induced ICAM-1 expression at its highest level at 360 minutes on the cell membrane. The cell viability assay indicated that cell viability of HUVECs showed no significant change after IL-1*β* treatment for 360 minutes in comparison to that of the control group (Suppl. Fig. [Supplementary-material supplementary-material-1]).

In order to further confirm that IL-1*β* could enhance MSC cell adhesion ability, a cell adhesion assay was performed on MSCs pretreated with IL-1*β* inhibitor IL-1RA for 2 hours and then cotreated with IL-1*β* for 30 minutes. The results showed that IL-1RA significantly suppressed IL-1*β*-induced MSC adhesion with activated HUVECs in comparison with the IL-1*β* treatment group (Figures 2(b) and 2(c)). However, IL-1RA treatments yielded no significant effect on inhibiting MSC adhesion in coculture with nonactivated HUVECs.

To examine whether IL-1*β*-induced MSC adhesion ability to HUVECs was affected through LFA-1/ICAM-1 interaction, the LFA-1 inhibitor lovastatin was used. A cell adhesion assay was performed to investigate the cell-cell adhesion ability between MSCs and HUVECs. The IC50 value of lovastatin on MSC adhesion ability is 50 *μ*M (Suppl. Fig. [Supplementary-material supplementary-material-1]). The cell adhesion assay was conducted after MSCs were pretreated with IL-1*β*, labelled with calcein AM, and then cocultured for 30 minutes with HUVECs which were untreated or treated with IL-1*β* for 6 hours. In order to investigate the role of LFA-1/ICAM-1 interaction, we added lovastatin which blocked the interaction in MSCs cocultured with HUVECs. When both cells were treated with IL-1*β*, the adhesion rate significantly increased when compared to the control group. Moreover, the adhesion cells were reduced significantly in the lovastatin treatment group (HUVECs with/without IL-1*β* treatment) (Figures 2(d) and 2(e)). The cell viability assay showed no significant change in MSCs and HUVECs after lovastatin treatment for 30 minutes when compared to the control group (Suppl. Fig. [Supplementary-material supplementary-material-1]). When both cells were treated with IL-1*β*, the adhesion rate significantly increased when compared to the control group. Moreover, the adhesion cells were reduced significantly in the lovastatin treatment group (HUVECs with/without IL-1*β* treatment).

### 3.3. p38 MAPK Signaling Pathway Is Involved in IL-1*β*-Mediated LFA-1 Expression in MSCs

**Figure 3:**
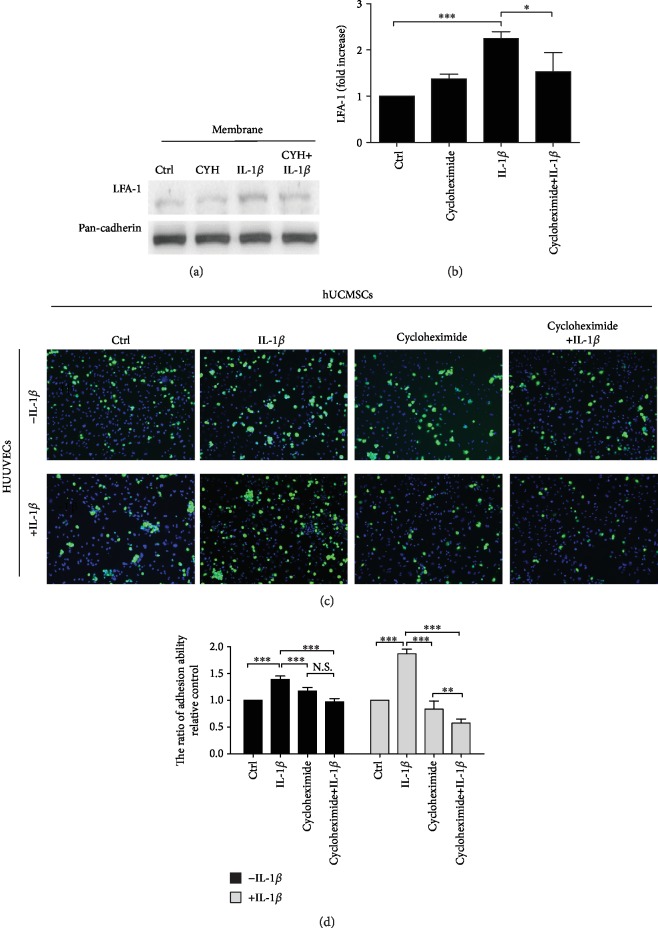
Effects of cycloheximide in MSC adhesion to HUVECs. (a) Western blot results of the LFA-1 (129 kDa) from membrane protein. Cells treated with IL-1*β* and cycloheximide (CYH) or cotreated with both IL-1*β* and CYH. (b) Quantitative graphs of the Western blot results of LFA-1 protein expression of (a). (*n* = 3, ^∗∗∗^*P* < 0.01, and ^∗^*P* < 0.05). (c) Representative image of MSC adhesion on HUVECs. MSCs treated with IL-1*β*; cycloheximide adhesion to IL-1*β*-activated HUVECs or nonactivated HUVECs. MSCs labelled with calcein AM (5 *μ*M) (green), HUVECs, and MSC cell nuclei were stained with Hoechst 33258 (blue) (scale bar: 50 *μ*m). (d) Quantitative graphs of the cell adhesion assay results of MSC adhesion to nonactivated HUVECs (black bars) or IL-1*β*-activated HUVECs (gray bars). MSCs treated with IL-1*β* and cycloheximide. MSC adhesion cell number counted in five randomly selected fields in a single representative experiment performed three times. Values were the cell number fold change relative to the control group. Data represent mean ± SD (*n* = 3, ^∗∗∗^*P* < 0.005, and ^∗∗^*P* < 0.01) (N.S.: nonsignificance).

In order to investigate LFA-1 protein expression through protein synthesis or translocation induced by IL-1*β* in MSCs, cells were pretreated with the protein synthesis inhibitor cycloheximide (20 *μ*g/ml) for 1 hour and then cotreated with IL-1*β* for 30 minutes. The result showed that cycloheximide suppressed IL-1*β*-induced LFA-1 protein level expression on the cell membrane (Figures 3(a) and 3(b)) and reduced IL-1*β*-induced MSC adhesion ability to HUVECs (Figures 3(c) and 3(d)).

### 3.4. The Role of p38 MAPK Pathway on IL-1*β*-Mediated MSC Adhesion to HUVECs

**Figure 4:**
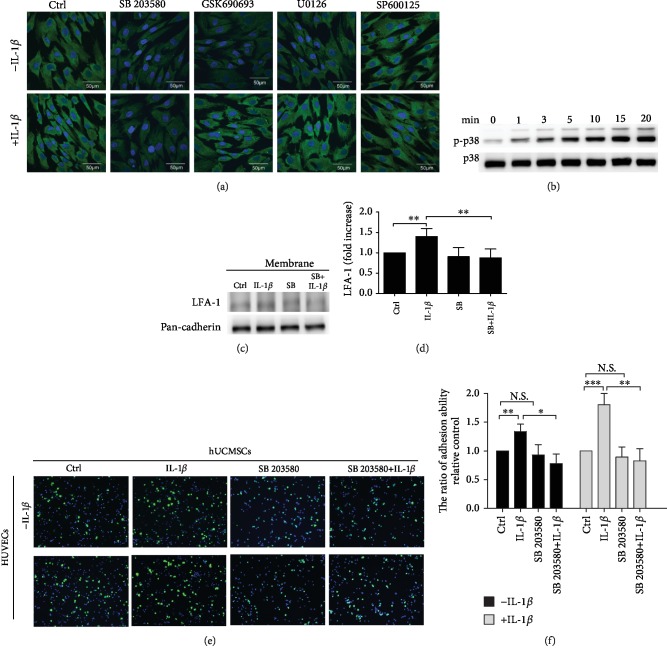
p38 MAPK signaling pathway is involved in IL-1*β*-mediated LFA-1 expression in MSCs and MSC adhesion to HUVECs. (a) Immunocytochemistry staining for LFA-1 (green) and DAPI (blue) in MSCs. Cells were treated with p38 MAPK (SB 203580, 5 *μ*M), AKT (GSK690693, 20 *μ*M), ERK1/2 (U0126 20 *μ*M), and JNK (SP600125 20 nM) inhibitor and combined with IL-1*β* for 30 minutes (scale bar: 50 *μ*m). (b) Western blot results of the p38 MAPK and phosphorylated p38 MAPK from the lysates of cells pretreated with IL-1*β* at 0, 1, 3, 5, 10, and 20 minutes. (c) Western blot results of the LFA-1 (129 kDa) expression in membrane fractions of MSCs treated with IL-1*β* and SB 203580 (SB) or cotreated with both IL-1*β* and SB. (d) Quantitative graphs of the Western blot results of LFA-1 expression of (c) (*n* = 3, ^∗∗^*P* < 0.01). (e) Representative image of MSC adhesion on HUVECs. MSCs were treated with IL-1*β* and inhibitor SB 203580 adhesion to IL-1*β*-activated HUVECs or nonactivated HUVECs. MSCs labelled with calcein AM (5 *μ*M) (green), HUVECs, and MSC cell nuclei were stained with Hoechst 33258 (blue) (scale bar: 50 *μ*m). (f) Quantitative graphs of the cell adhesion assay results of MSCs treated with IL-1*β* and MAPK inhibitor SB 203580 adhesion to nonactivated HUVECs (black bars) or IL-1*β*-activated HUVECs (gray bars). Values were the cell number fold change relative to the control group. Data represent mean ± SD (*n* = 3, ^∗∗∗^*P* < 0.005, ^∗∗^*P* < 0.01, and ^∗^*P* < 0.05) (N.S.: nonsignificance).

To observe whether p38 MAPK, AKT, ERK1/2, and JNK signaling pathways in the IL-1*β*-induced MSC cell membrane impact LFA-1 protein expression, we performed immunocytochemistry staining for LFA-1 in MSCs. MSCs were treated with the p38 MAPK inhibitor SB 203580 (5 *μ*M), AKT inhibitor GSK690693 (20 *μ*M), ERK1/2 inhibitor U0126 (20 *μ*M), and JNK inhibitor SP600125 (20 nM) and then combined with IL-1*β* for 30 minutes. The results showed that the p38 MAPK inhibitor SB 203580 inhibited LFA-1 expression in both non-IL-1*β*-induced and IL-1*β*-induced MSCs (Figure 4(a)). We also found that the AKT inhibitor GSK690693 and ERK1/2 inhibitor U0126 only inhibited LFA-1 expression in non-IL-1*β*-induced, but not IL-1*β*-treated, MSCs. Western blot showed that MSCs pretreated with IL-1*β* induced p38 MAPK phosphorylation (Figure 4(b)), suggesting that IL-1*β* induced the p38 signaling pathway. We further confirmed that the p38 MAPK pathway in the IL-1*β*-induced MSC cell membrane affects LFA-1 protein expression. Results showed that IL-1*β* cotreated with SB 203580 significantly reduced the protein expression when compared to the IL-1*β* treatment group (Figures 4(c) and 4(d)).

To further confirm the role of the p38 MAPK pathway in IL-1*β*-induced MSC adhesion ability, a cell adhesion assay was performed in MSCs cotreated with IL-1*β* and the p38 MAPK inhibitor SB 203580. MSCs were treated with the inhibitor SB 203580 (5 *μ*M) or cotreated with SB 203580 and IL-1*β* for 30 minutes (Figures 4(e) and 4(f)). Data showed that inhibitor SB 203580 did not affect MSC adhesion ability in comparison with the control group. But cotreated SB 203580 with IL-1*β* significantly suppressed MSC adhesion to activated HUVECs when compared to the IL-1*β* treatment group. Furthermore, MSC adhesion in nonactivated HUVECs exhibited the same trend. The cell viability assay indicated that MSC cell viability showed no significant change after treatment with SB 203580 for 30 minutes when compared to the control group (Suppl. Fig. [Supplementary-material supplementary-material-1]).

## 4. Discussion

Mesenchymal stem cells (MSCs) are well known for their ability to regenerate injured tissue [33]. Currently, there are two delivery methods in MSC therapy: direct local implantation or systemic intravascular administration. Previous studies found that MSCs tend to die in circulation without leaving vessels [6] or become trapped in unwanted organs [34] after their intravenous injection into the body. Only 1% of MSCs are able to find their way to the target tissues [35–37]. In order for MSC therapy to be efficacious, investigation into mechanisms of MSC homing is essential.

There are three steps in the homing mechanism: rolling, adhesion, and transmigration. In this study, we focused on the cell adhesion step because many reports have shown that cell-endothelial cell-adhesive interactions compared between cell adhesion molecules may provide further insight into the potential mechanisms of MSC homing [38, 39]. According to Segers et al., MSCs and cardiac microvascular endothelial cells activated with TNF-*α* or IL-1*β* before adhesion can increase MSC adhesion to endothelial cells [40].

In our research, we speculated that IL-1*β* induced MSC and HUVEC cell-adhesive interactions. Previous studies demonstrated that VLA-4/VCAM-1 adhesive interactions of MSCs form adhesion to endothelial cells [15, 41]. Ko et al. found that MSCs coated with palmitated protein G (PPG) followed by treatment with antibodies against ICAM-1 promoted MSC attachment to endothelial cells [42]. ICAM-1 expression on endothelial cells has been detected during the MSC adhesion step, but it is not known which ligands are present in MSC interaction with this receptor [6]. The LFA-1/ICAM-1 receptor-ligand pair is central to leukocyte-initiated adhesion to endothelial cells [43]. Taken together, we hypothesized that MSCs and HUVECs treated with IL-1*β* would induce LFA-1/ICAM-1 cell-adhesive interactions. In our study, we used Western blotting and immunofluorescence staining to examine IL-1*β*-induced LFA-1 expression on MSCs (Figure 1) and IL-1*β*-induced ICAM-1 expression on HUVECs (Figure 2). In contrast, HUVECs stimulated with IL-1*β* did not induce LFA-1 expression (Suppl. Fig. [Supplementary-material supplementary-material-1]). MSCs stimulated with IL-1*β* did not induce ICAM-1 expression (Suppl. Fig. [Supplementary-material supplementary-material-1]). The in vitro study cell adhesion assay showed that the LFA-1 antagonist lovastatin inhibits MSC adhesion to HUVECs. Lovastatin is able to inhibit LFA-1/ICAM-1 interaction in vitro by binding to the LFA-1 L-site. Lovastatin does not bind to L-site-like motifs in other I domains such as *β*2 integrin Mac-1 (also known as CD11b/CD18) [44, 45]. Previous research showed that lovastatin blocking LFA-1/ICAM-1 interaction can decrease T-cell activation. This could be a potential therapy for inflammation and immunosuppression [46]. Our results showed that lovastatin can inhibit LFA-1/ICAM-1 cell-adhesive interactions when cocultured with MSCs and HUVECs (Figure 2(d)). Moreover, activation by IL-1*β* followed by treatment with lovastatin significantly suppresses MSC adhesion to HUVECs. With these results, we prove that IL-1*β* promotes MSC adhesion to HUVECs through upregulation of LFA-1/ICAM-1 cell-adhesive interactions. Interestingly, IL-1*β*-induced MSC adhesion to nonactivated HUVECs did not significantly enhance the MSC adhesion ability. This result does not surprise us because we found high expressions of ICAM-1 in HUVECs treated with IL-1*β* for 6 hours in comparison with the control group (Figure 2(a)). From research into acute inflammation, we know that endothelial cell activation is mediated by inflammatory factors in damaged tissue. MSCs target activated endothelial cells through blood flow to damaged tissue. Cell-endothelial cell adhesion is induced by inflammatory factors in a very short time period [47, 48]. From our results, we confirmed that the presence of IL-1*β* was required initially only for a short time to induce LFA-1/ICAM-1 cell-adhesive interactions of MSCs on HUVECs.

Based on the findings presented in this work, IL-1*β* induces MSCs with the ability to upregulate LFA-1 expression and enhance cell adhesion ability in a very short period of time. To determine whether these effects required new protein synthesis, MSCs were pretreated with cycloheximide, a translational inhibitor, before IL-1*β* treatment. We found that treatment with cycloheximide attenuates LFA-1 protein expression and cell adhesion ability. These results suggest that new protein synthesis was required. Next, we wanted to further investigate the downstream mediators responsible for IL-1*β*-induced LFA-1 expression. IL-1*β* triggers the IL-1 pathway downstream signaling molecule nuclear factor kappa B (NF*κ*B) and MAPK (p38, JNK, and ERK) pathways. Previous studies demonstrated that p38 MAPK is a major mediator in IL-8-activated LFA-1, Mac-1, and *α*4-integrin in the neutrophil [49]. Our results (Figure 4(a)) showed that in immunocytochemistry staining of IL-1*β* cotreated with inhibitors (p38 MAPK, JNK, ERK1/2, and Akt), only the p38 MAPK inhibitor affects IL-1*β*-induced LFA-1 expression. These results demonstrate that the p38 MAPK inhibitor significantly blocks LFA-1 expression. Furthermore, we examined protein levels of LFA-1 affected by cotreatment of IL-1*β* with the p38 MAPK inhibitor and cell adhesion assay to determine the role of p38 MAPK in cell-endothelial cell adhesion mechanism (Figure 4). In Results, we show that IL-1*β* induce the LFA-1 protein expression path through the p38 MAPK pathway in MSCs. Moreover, p38 MAPK inhibitor treatment did not affect MSC adhesion to HUVECs, but cotreatment of IL-1*β* with the p38 MAPK inhibitor significantly decreases MSC adhesive ability. Previous results confirm the crucial role of p38 MAPK in the IL-1*β*-induced IL-1 pathway.

## 5. Conclusions

**Figure 5:**
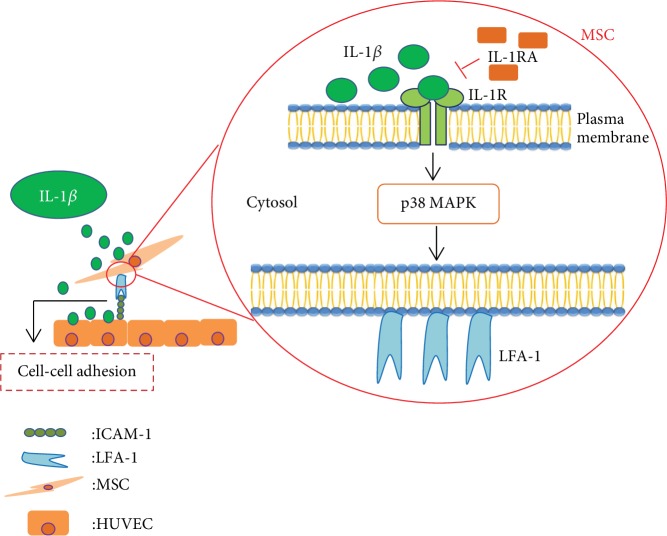
Schematic diagram of IL-1*β* signaling pathway in MSC adhesion to HUVECs. The schematic diagram depicts the proposed role of IL-1*β* signaling pathway in MSC adhesion to HUVECs. The process of cell adhesion is initiated by IL-1*β* through p38 MAPK; induced expression of LFA-1 in MSCs enhances the cell adhesion to IL-1*β*-induced ICAM-1 in HUVECs.

In conclusion, the results of this study show that IL-1*β* promotes LFA-1/ICAM-1 cell-adhesive interactions of MSC adhesion to endothelial cells and further indicates that IL-1*β* induces LFA-1 expression in MSCs through p38 MAPK (Figure 5). This study demonstrates a new strategy to improve therapeutic efficacy of cell-based therapies by enhancing MSC adhesion to endothelial cells prior to homing to sites of inflammation.

## Data Availability

The data used to support the findings of this study are included within the article.

## Supplementary Materials

Supplementary Materials Supplementary Figure 1: cell viability assay for drug cytotoxicity impact on MSCs and HUVECs. (A) MSCs treated with IL-1*β* for 30 minutes, IL-1RA for 150 minutes, lovastatin for 30 minutes, SB 203580 for 30 minutes, cycloheximide for 90 minutes, and DMSO for 90 minutes. (B) HUVECs treated with IL-1*β* for 6 hours, lovastatin for 30 minutes, and DMSO for 30 minutes. Data were quantified by multimode microplate readers. Data are shown as the mean ± SD (*n* = 3). (N.S.: nonsignificance). Supplementary Figure 2: different concentrations of lovastatin inhibit IL-1*β*-induced MSC adhesion to HUVECs; IL-1*β*-induced MSCs to HUVECs were inhibited by lovastatin at various concentrations. Data represent mean ± SD (*n* = 3) (^∗∗∗^*P* < 0.005, ^∗∗^*P* < 0.01, and ^∗^*P* < 0.05). Supplementary Figure 3: the expression of LFA-1 in HUVECs pretreated with IL-1*β* and HUVECs without treated IL-1*β* as a control group or treated with IL-1*β* for 15, 30, 120, and 360 minutes. Immunocytochemistry staining for LFA-1 (green) and DAPI (blue) in HUVECs. Supplementary Figure 4: the expression of ICAM-1 in MSCs pretreated with IL-1*β* MSCs without treated IL-1*β* as a control group or treated with IL-1*β* for 15, 30, 120, and 360 minutes. Immunocytochemistry staining for ICAM-1 (green) and DAPI (blue) in MSCs.
