# Supplementary figures and images for: Interleukin-1β Enhances Umbilical Cord Mesenchymal Stem Cell Adhesion Ability on Human Umbilical Vein Endothelial Cells via LFA-1/ICAM-1 Interaction
Source: Stem Cells Int. 2019 Dec 27;2019:7267142. doi: 10.1155/2019/7267142 (PMC6948307; doi:10.1155/2019/7267142)

Supplementary figure 1

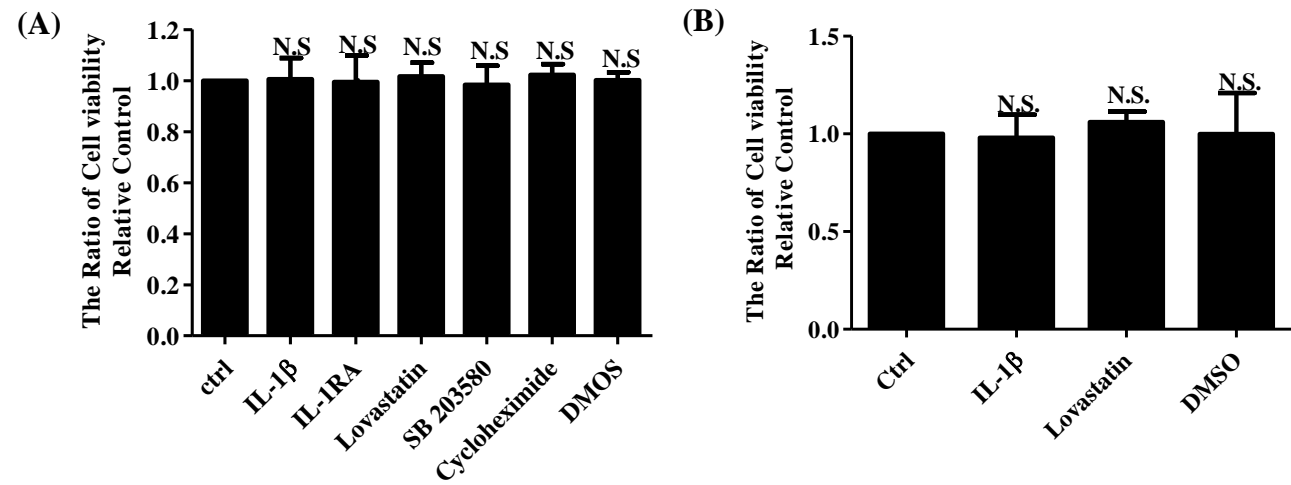

Supplementary figure 2

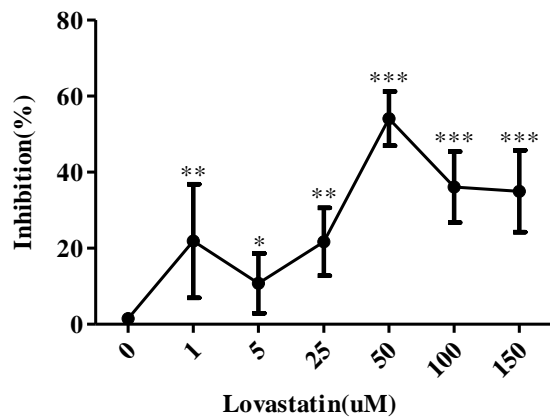

Supplementary figure 3

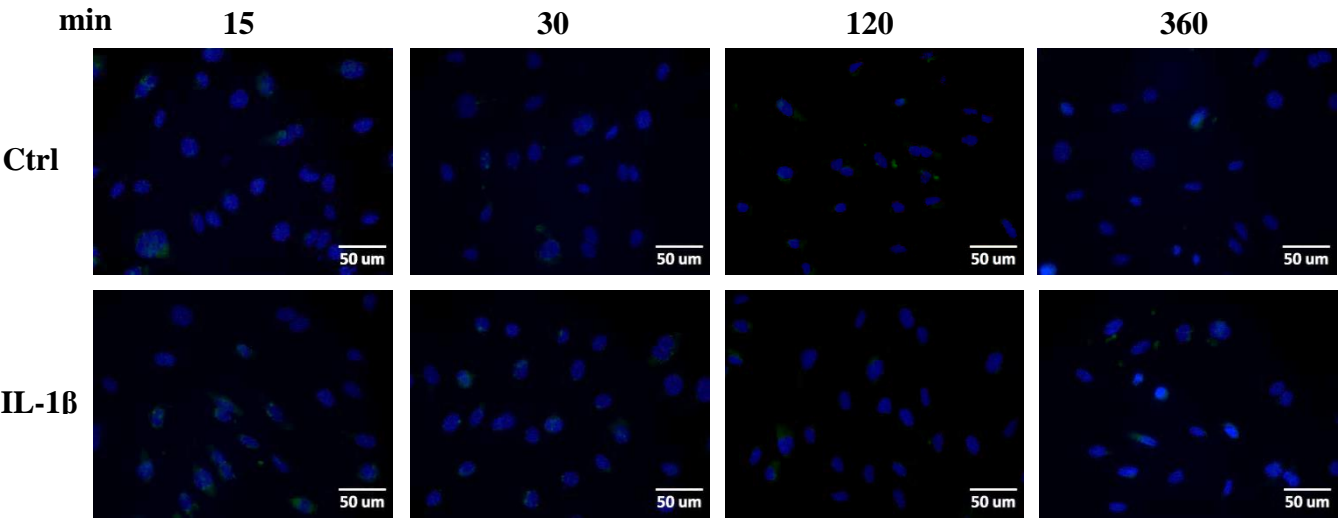

Supplementary figure 4

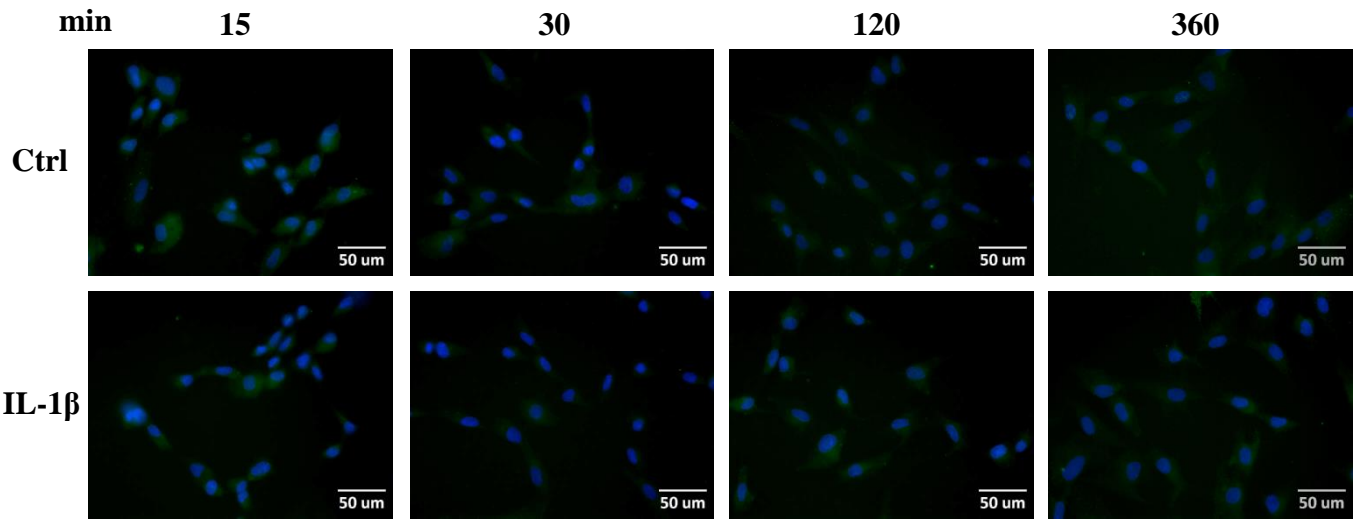

Supplement: Supplementary Materials — Supplementary Figure 1: cell viability assay for drug cytotoxicity impact on MSCs and HUVECs. (A) MSCs treated with IL-1β for 30 minutes, IL-1RA for 150 minutes, lovastatin for 30 minutes, SB 203580 for 30 minutes, cycloheximide for 90 minutes, and DMSO for 90 minutes. (B) HUVECs treated with IL-1β for 6 hours, lovastatin for 30 minutes, and DMSO for 30 minutes. Data were quantified by multimode microplate readers. Data are shown as the mean ± SD (n = 3). (N.S.: nonsignificance). Supplementary Figure 2: different concentrations of lovastatin inhibit IL-1β-induced MSC adhesion to HUVECs; IL-1β-induced MSCs to HUVECs were inhibited by lovastatin at various concentrations. Data represent mean ± SD (n = 3) (∗∗∗P < 0.005, ∗∗P < 0.01, and ∗P < 0.05). Supplementary Figure 3: the expression of LFA-1 in HUVECs pretreated with IL-1β and HUVECs without treated IL-1β as a control group or treated with IL-1β for 15, 30, 120, and 360 minutes. Immunocytochemistry staining for LFA-1 (green) and DAPI (blue) in HUVECs. Supplementary Figure 4: the expression of ICAM-1 in MSCs pretreated with IL-1β MSCs without treated IL-1β as a control group or treated with IL-1β for 15, 30, 120, and 360 minutes. Immunocytochemistry staining for ICAM-1 (green) and DAPI (blue) in MSCs. [file 7267142.f1.pdf]
